# A Year at the Forefront of Bacterial Defense Systems Against Neutrophilic Oxidants

**DOI:** 10.1242/bio.059809

**Published:** 2023-04-27

**Authors:** Sadia Sultana, Jan-Ulrik Dahl

**Affiliations:** School of Biological Sciences, Microbiology, Illinois State University, Campus Box 4120, Normal, IL 71790, USA

**Keywords:** Bacterial defense systems, Hypohalous acids, Oxidative stress, Redox regulation, Stress response, Transcriptional regulators

## Abstract

One challenge for invading pathogens represents the exposure to highly microbicidal hypohalous acids (HOX), such as hypochlorous acid (HOCl) and hypothiocyanous acid (HOSCN). Generated at high concentrations by innate immune cells during phagocytosis, HOX kills the engulfed microbes through extensive macromolecular damage. However, microorganisms have evolved strategies to detoxify the oxidants and/or alleviate HOX-mediated damage, which improves their survival during HOX exposure. Many of these defense systems are bacteria-specific and therefore considered potential drug targets. Our minireview highlights recent (July 2021 to November 2022) advances in the field of microbial HOX defense systems and how these systems are regulated. We report recent progress on redox-sensing transcriptional regulators, two-component systems, and σ/anti-σ factors and review how oxidative modifications in these regulatory proteins affect the expression of their target genes. Moreover, we discuss novel studies that describe how HOCl affects the activity of redox-regulated enzymes and highlight mechanisms that bacteria employ to reduce HOSCN.

## Teaser

**Summary:** This a Year at the Forefront Review sheds light on the most recent developments in the field of bacterial responses and defenses against reactive oxygen and chlorine species

## Introduction

The cellular imbalance between the production and accumulation of reactive oxygen and chlorine species (ROS/RCS) and antioxidant defenses is a phenomenon called oxidative stress. In fact, ROS/RCS accumulate during inflammation and appear to be involved in controlling bacterial colonization of epithelia, where they are generated by dual oxidases (El Hassani et al., 2005; Bae et al., 2010). Moreover, innate immune cells, such as neutrophils and macrophages, produce high levels of ROS/RCS to kill invading pathogens in a process called phagocytosis (Winterbourn et al., 2016; Winterbourn and Kettle, 2013; Klebanoff et al., 2013). During respiratory burst, NADPH oxidases are assembled on the phagosomal membrane to catalyze the reduction of molecular oxygen to superoxide, which is subsequently dismutated to hydrogen peroxide (H_2_O_2_) and released into the phagosomal space (Hampton et al., 1998). The release of myeloperoxidase into the phagosome catalyzes the conversion of the accumulating H_2_O_2_ and available (pseudo-) halides (i.e. Cl^−^, Br^−^, and SCN^−^) into hypohalous acids (HOX), such as hypochlorous acid (HOCl), hypobromous acid (HOBr), and hypothiocyanous acid (HOSCN), respectively (Winterbourn et al., 2016; Hurst, 2012; Davies, 2011).

HOX are extremely reactive and bactericidal already at low micromolar levels (Nagl et al., 2000; Love et al., 2016). A common target of all neutrophilic oxidants is the amino acid cysteine (Winterbourn and Kettle, 2013; Winterbourn et al., 2016). HOX oxidize cysteines to either reversible (i.e. sulfenic acids; disulfide bonds) or irreversible thiol modifications (i.e. sulfinic and sulfonic acid) (Dahl et al., 2015). Reversible thiol modifications often come along with severe structural and functional consequences, while irreversible thiol modifications can lead to protein aggregation and degradation (Dahl et al., 2015; Cremers and Jakob, 2013). A study in *Pseudomonas aeruginosa* revealed overlapping outcomes for treatments with HOCl and HOBr as both oxidants target non-growing cells more efficiently than growing cells and elicit similar bacterial responses (Groitl et al., 2017). Exposure to HOCl causes pleiotropic phenotypes in bacterial cells given that this oxidant can oxidize and damage virtually any cellular molecule, including select amino acids, lipids, metal centers, and nucleic acids (Gray et al., 2013a). These oxidative modifications can cause protein aggregation, DNA strand cleavage, mis-metalation, ATP depletion, and a substantial reduction in the free thiols pool, ultimately leading to microbial death. In contrast, treatment with HOSCN has been found to affect primarily actively growing cells and evoking different defense mechanisms (Groitl et al., 2017), likely due to its highly thiol-specific nature (Skaff et al., 2009).

However, bacteria have likewise evolved mechanisms to counter the detrimental effects of HOX (recently reviewed in Gray et al., 2013a; Dahl et al., 2015; Sultana et al., 2020; Varatnitskaya et al., 2021; Ulfig and Leichert, 2021; Aussel and Ezraty, 2021). Notably, microorganisms mount responses to changes in their environment, such as the exposure to HOX, on both transcriptional and post-translational level. Our review therefore highlights the most recent advances in the area of bacterial defense systems against the neutrophilic oxidants.

## A year at the forefront of bacterial defense systems against neutrophilic oxidants

### Discoveries

#### Redox-regulated transcription factors

Microbial responses to ROS/RCS often involve redox-sensitive transcriptional regulators, which use conserved cysteine and/or methionine residues to modulate their activity (Gray et al., 2013a). This, in turn, upregulates the transcription of their target genes, many of which have been shown to protect the organism from ROS/RCS. Three HOCl-responsive transcriptional regulators have been identified in *E. coli* prior to 2021, all of them in the K12-strain MG1655: (1) HypT (Gebendorfer et al., 2012); (2) the TetR-family transcriptional repressor NemR (Gray et al., 2013b), and (3) the AraC-family transcriptional activator RclR (Königstorfer et al., 2021; Parker et al., 2013).

**Fig. 1.**
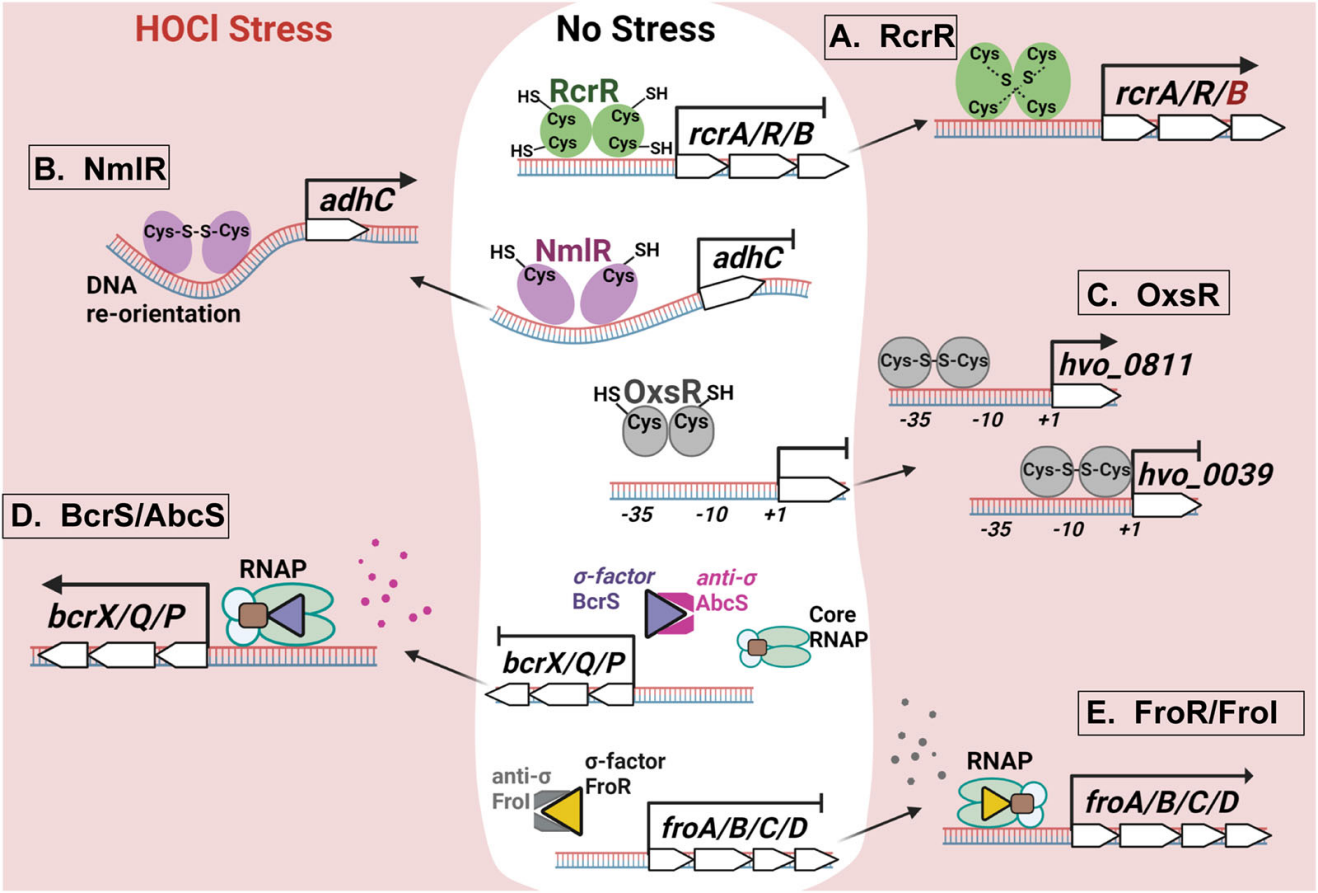
**Exposure to HOCl causes substantial transcriptional changes in microorganisms, which are mediated by redox-sensing transcriptional regulators, two-component systems, and σ factors.** (A) The transcriptional repressor RcrR forms reversible intermolecular disulfide bonds upon HOCl-stress resulting in its dissociation from the operator and derepression of the *rcrARB* genes. Expression of RcrB protects uropathogenic *E. coli* from HOCl-stress *in vitro* and contributes to increased resistance during phagocytosis. (B) *Streptococcus pneumoniae* NmlR is a transcriptional activator that remains bound to promoter region under both non-stress and HOCl-stress conditions. NmlR forms intermolecular disulfide bonds in the presence of HOCl, which may distort the DNA, improve RNA polymerase binding, and thus increase transcription of *adhC* to presumably detoxify HOCl by an unknown mechanism. (C) The archaeon *H. volcanii* employs the transcriptional regulator OxsR to protect itself from the deleterious effects of HOCl. OxsR can function as transcriptional activator and repressor, which is dictated by the position of the GC-rich binding site in the promoter region. (D) In *Brucella* sp., transcription of the *bcrXQP* genes that encode a methionine-rich peptide, and a methionine sulfoxide reductase homolog is controlled by the σ/anti-σ factors BcrS/AbcS. Under non-stress conditions, AbcS binds BcrS and reduces its interaction with RNA polymerase, resulting in low transcriptional outcomes. HOCl proteolytically cleaves AbcS and increases BcrS availability for RNA polymerase resulting in elevated *bcrXQP* transcription. (E) The σ/anti-σ-sigma factors FroR/FroI regulate transcription of the *P. aeruginosa froABCD* operon by an unknown mechanism. During HOCl-stress, the presence of FroR is required for efficient upregulation of *froABCD*, which provide protection from HOCl.

Sultana et al. reported that uropathogenic *E. coli* (UPEC) are substantially more resistant to HOCl and killing by neutrophils due to the presence of an additional HOCl-defense system that intestinal *E. coli* lack (Sultana et al., 2022). The TetR-family transcriptional repressor RcrR is reversibly inactivated through HOCl-mediated cysteine oxidation leading to the de-repression of transcription of the *rcrARB* operon (Fig. 1A). HOCl causes the formation of intermolecular disulfide bonds in RcrR, which results in conformational changes and contributes to RcrR's dissociation from the promoter. UPEC's increased HOCl resistance appears to be exclusively mediated by RcrB, a putative inner membrane protein of unknown function, as *rcrB*-deficient UPEC strains were similarly susceptible to HOCl as the HOCl-sensitive intestinal *E. coli* strains tested (Sultana et al., 2022).

HOCl-sensing transcription factors also play an important role for the activation of HOCl defense systems in Gram-positive pathogens (Beavers and Skaar, 2016)*.* A recently studied example is the *Streptococcus pneumoniae* 1-Cys-type regulator NmlR, which forms intermolecular disulfide bonds upon oxidation of its redox-sensitive cysteine residue but remains bound to the promoter DNA regardless of its oxidation state (Fritsch et al., 2023). Oxidized NmlR presumably distorts the promoter DNA, resulting in improved RNA polymerase binding and increased *adhC* transcript levels (Fig. 1B)*.* Not surprisingly, growth of the *nmlR* and *adhC* deletion strains were significantly impaired during exposure to sublethal HOCl concentrations or in macrophages. However, how AdhC, a class III alcohol dehydrogenase, confers resistance to HOCl is still unclear.

Within the last year, an additional HOCl-defense system has been identified in the haloarchaeal species *Haloferax volcanii*: The TrmB-family regulator OxsR interacts with distinct intergenic regions of the *H. volcanii* genome to control transcription of numerous downstream genes, including antioxidant genes and Fe/S-cluster-containing proteins (Mondragon et al., 2022). Transcriptional analyses of randomly selected downstream genes revealed OxsR's versatile regulatory nature as the protein can act as an activator or repressor depending on the presence and location of a GC-rich binding motif (Fig. 1C). The authors proposed that formation of an intermolecular disulfide bond results in homodimer formation upon oxidation with HOCl, and triggers OxsR binding to the promoter.

#### HOCl-responsive two-component systems

Other stress responses are governed by the action of two-component systems (TCS): histidine kinases sense and transmit the incoming signal to a response regulator, which executes the output response upon phosphorylation by the histidine kinase (Breland et al., 2017). H_2_O_2_ was the first signal, which activates the *E. coli* TCS HprSR, causing the upregulation of the *msrPQ* genes (Urano et al., 2015). *msrPQ* encode a methionine sulfoxide reductase, which consists of the molybdopterin-containing oxidoreductase MsrP and the heme-containing membrane protein MsrQ (Gennaris et al., 2015). MsrP repairs oxidized proteins in the periplasm by converting methionine sulfoxides to methionine residues. A recent study by Hajj et al. found that HOCl and the related compound *N*-chlorotaurine represent a more efficient activation signal than other thiol-reactive compounds, including H_2_O_2,_ diamine, paraquat, and nitric oxide (El Hajj et al., 2022). Two methionine residues present in the periplasmic loop of HprS were identified to be responsible for the sensing activity, whereas a cysteine residue in the transmembrane region is important for signal transduction (El Hajj et al., 2022).

#### HOCl-controlled σ/anti-σ factor interaction

To initiate RNA polymerase binding to the promoter and start transcription, RNA polymerase requires a σ factor, which occasionally is co-expressed with its cognate anti-σ factor (Saecker et al., 2011). Anti-σ factors bind their cognate (extracytoplasmic function) ECF σ factors with high affinity and specificity to prevent the formation of holo-RNA polymerase. However, induced by extracellular signals, some ECF σ factors rely on proteolytic cleavage of the anti-σ factor, which increases the cellular amount of σ factor and results in increased transcription (Helmann, 2002). Over the last year, two σ/anti-σ factor systems have been identified that respond to HOCl-stress.

The *Brucella melitensis bcrS*/*abcS* system controls the expression of the *bcrXQP* operon, which encodes a methionine-rich peptide and a homolog of *E. coli* methionine sulfoxide reductase MsrPQ (Li et al., 2021). AbcS presumably binds BcrS under non-stress conditions and reduces its interaction with RNA polymerase (Fig. 1D). Under HOCl-stress, however, AbcS is proteolytically cleaved and the σ factor BcrS is released and accumulates, which results in increased *bcrXQP* transcription. Moreover, BcrS was also shown to induce the expression of a type IV secretion system; however, this appeared to be independent of the anti-σ factor AbcS (Li et al., 2021). Interestingly, the Δ*bcrS* strain showed wild-type-like survival in an *in vivo* mouse model, suggesting that BcrXQP expression has no significant protective role during infection, which, however, contrasts with other studies (Juillan-Binard et al., 2017; Jalal and Lee, 2020; Beavers et al., 2021; Tossounian et al., 2020).

In *P. aeruginosa*, expression of the *froABCD* operon improves their survival in the highly oxidizing environment of the neutrophil phagosome (Foik et al., 2022 preprint). All four members of the operon are uncharacterized proteins; however, FroA and FroB are predicted to be cytoplasmic while FroC and FroD are putative inner membrane proteins. Transcription of *froABCD* appears to be controlled by the σ/anti-σ factor system FroR/FroI by an unknown mechanism (Foik et al., 2022 preprint). The pronounced growth defect of a Δ*froR* mutant during HOCl-stress, its reduced ability to express antioxidant proteins, and previous induction studies under flow conditions suggest that HOCl-induced *froABCD* expression requires the presence of FroR and absence of FroI (Fig. 1E) (Foik et al., 2022 preprint; Sanfilippo et al., 2019).

#### HOCl-mediated changes in protein activity

**Fig. 2.**
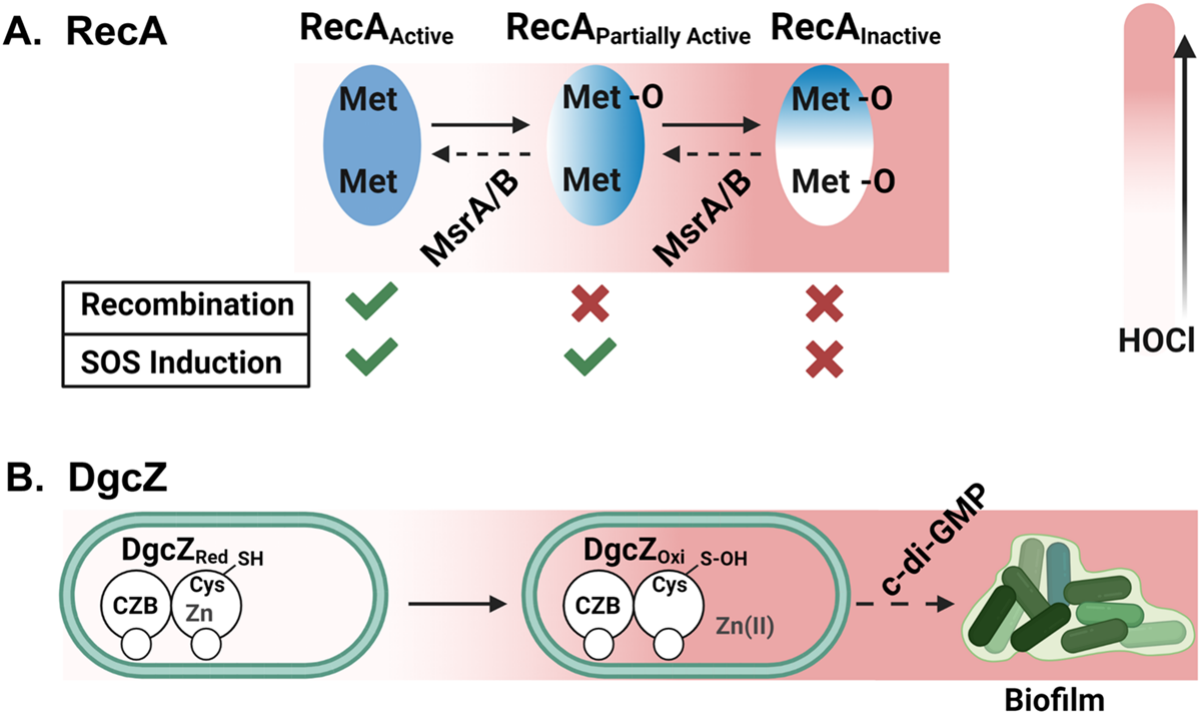
**Oxidation of redox-sensitive amino acids by HOCl affect the catalytic activity of enzymes.** (A) H_2_O_2_-mediated oxidation of conserved methionine residues result in RecA's partial and/or full inactivation, which negatively affects homologous recombination and induction of the SOS response. RecA oxidation can be reversed by the methionine sulfoxide reductase MsrAB. (B) HOCl-mediated cysteine oxidation of the *E. coli* diguanylate cyclase DgcZ causes the disruption of the zinc-thiolate complex, resulting in an increased enzymatic activity. As a result, c-di-GMP production increases and promotes biofilm formation.

Proteins constitute for >50% of the cellular macromolecules and are known to rapidly react with HOCl (Hawkins and Davies, 2019). Numerous studies in different HOCl-treated bacterial species revealed the strong upregulation of the heat shock regulon, indicating an accumulation of misfolded proteins and supporting the idea that proteins are the major targets of HOCl (Groitl et al., 2017; Gray et al., 2013b; Sultana et al., 2022; Thakur et al., 2019; Tung et al., 2020; Hillion et al., 2017). Similarly, H_2_O_2_ can cause substantial protein aggregation as the result of methionine and cysteine oxidation (Imlay, 2008). One recently identified target of HOCl/ H_2_O_2_ is the ubiquitous DNA recombination/repair protein RecA, a crucial member of the RecBCD-dependent DNA damage repair system (Henry et al., 2021). Both HOCl and H_2_O_2_ inactivate RecA through the oxidation of at least two conserved methionine residues into methionine sulfoxides, although oxidation by HOCl was more pronounced likely due to its higher potency. Oxidized RecA was unable to form nucleoprotein filaments, showed little to no DNA-dependent ATPase activity, and no longer promoted DNA strand exchanges (Fig. 2A). However, incubation of oxidized RecA with the methionine sulfoxide reductase MsrAB restored, at least partially, its function (Henry et al., 2021).

In two independent studies, Perkins et al. reported the mechanisms for novel adaptive survival strategies stimulated by HOCl (Perkins et al., 2019; 2021). The most recent study focused on the HOCl-mediated increase in activity of the *E. coli* diguanylate cyclase DgcZ, an enzyme that generates the biofilm second messenger cyclic-dimeric-GMP (c-di-GMP). DgcZ contains an N-terminal chemoreceptor zinc-binding site (CZB) that is also present in the C-terminus of *Helicobacter pylori* TlpB, where it was shown to coordinate chemoattraction to HOCl (Perkins et al., 2019; 2021). Notably, *E. coli* utilizes the same structural topology of the CZB domain to regulate diguanylate cyclase activity for the production of c-di-GMP. In DgcZ, the CZB domain senses HOCl through reversible thiol oxidation of a conserved cysteine into cysteine sulfenic acid, resulting in conformational changes that negatively affects CZB's zinc-binding affinity. The release of zinc provides the DgcZ protein with more structural flexibility and allows the GGDEF domain to increase the production of c-di-GMP. The increased c-di-GMP level, in turn, positively affects the synthesis of poly-N-acetylglucosamine (poly-GlcNAc) (Perkins et al., 2021; Poulin and Kuperman, 2021), an exopolysaccharide essential for biofilm formation in various *E. coli* pathotypes (Boehm et al., 2009).

#### Detoxification of HOSCN by NAD(P)H-dependent reductases

Mammalian cells are well equipped to deal with the consequences of HOSCN-stress due to the presence of thioredoxin reductase, a selenocysteine-containing flavoprotein disulfide reductase that directly reduces HOSCN through oxidation of NADPH (Chandler et al., 2013). In contrast, the bacterial thioredoxin reductase homolog lacks the selenocysteine, has a narrower substrate range, and was even inhibited by HOSCN (Chandler et al., 2013). This was surprising given that certain bacterial species show increased resistance to HOSCN (Shearer et al., 2022a).

**Fig. 3.**
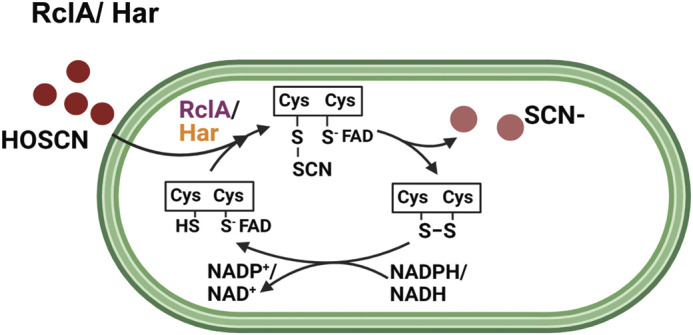
**HOSCN is detoxified by HOSCN reductases.** *Streptococcus pneumoniae* Har (orange) and *E. coli* RclA (purple) are flavin-containing HOSCN reductases. The N-terminal thiol group in Har/ RclA reacts with HOSCN to form a sulfenyl-thiocyanate intermediate, which is subsequently attacked by the C-terminal thiol group, resulting in disulfide bond formation and the release of SCN^−^. In both enzymes, the disulfide bonds can be reduced by NADH and/or NADPH.

However, independent studies by Shearer et al. and Meredith et al. identified the flavoproteins Har and RclA (Fig. 3) as efficient HOSCN reductases in *S. pneumoniae* and *E. coli*, respectively (Shearer et al., 2022b; Meredith et al., 2022). The enzymatic action of RclA follows a ping-pong kinetic mechanism, where the N-terminal cysteine thiol in the active site reacts with HOSCN to a sulfenyl-thiocyanate intermediate. The thiocyanate anion is released upon formation of a disulfide bond formation with the C-terminal thiol group. Subsequent reduction of RclA is mediated by the oxidation of NAD(P)H. Intriguingly, despite the homology of Har and RclA, both enzymes appear to elicit different phenotypes. While *rclA-*deficient *E. coli* cells showed a significant growth arrest in presence of HOSCN and RclA overexpression renders them highly resistant (Meredith et al., 2022), a *har*-deficient *S. pneumoniae* strain only appears more sensitive in the absence of the glutathione import and recycling system, which itself had been identified to protect the pathogen from HOSCN (Shearer et al., 2022b,c).

## Future prospects

Given the physiological significance of HOX exposure during infection, research in this field is rapidly evolving even though we are still far away from understanding the full picture of HOX defenses in bacteria. The bacterial response and defense strategies are expected to be critical for their ability to survive the immune cell attack, as reported by several recent studies (Sultana et al., 2022; Fritsch et al., 2023; Foik et al., 2022 preprint). Moreover, independent studies confirmed that the presence of functional oxidative stress defense systems positively affects pathogen colonization in the host, emphasizing their importance for pathogenesis (Peng et al., 2020; Dahl et al., 2017; Hryckowian and Welch, 2013; Bessaiah et al., 2019). Therefore, targeting processes that are essential for bacterial survival only in the context of infections and directly contribute to bacterial virulence and persistence represent intriguing alternative drug targets (Flores-Mireles et al., 2015; Moradali et al., 2017).
